# The effect of ponicidin on CFA-induced chronic inflammatory pain and its mechanism based on network pharmacology and molecular docking

**DOI:** 10.3389/fmed.2025.1510271

**Published:** 2025-02-19

**Authors:** Peng Wang, Huiyi Jiang, Jinzhong Yao, Guangting He, Tao Tao, Zaisheng Qin

**Affiliations:** ^1^Department of Anesthesiology, Nanfang Hospital, Southern Medical University, Guangzhou, Guangdong, China; ^2^The Key Laboratory of Precision Anesthesia and Perioperative Organ Protection, Guangzhou, Guangdong, China; ^3^Department of Anesthesiology, Zhujiang Hospital, Southern Medical University, Guangzhou, Guangdong, China

**Keywords:** ponicidin, pain, network pharmacology, molecular docking, *TLR4*, *HIF1A*

## Abstract

**Purpose:**

Inflammation is a frequent precursor to the development of chronic pain. Ponicidin, a compound derived from traditional Chinese medicine, possesses immunomodulatory and anti-inflammatory properties. However, whether ponicidin mitigates inflammatory pain through its anti-inflammatory effects and potential target molecules remains to be further explored. In this study, we investigated the analgesic effects of ponicidin in a mouse model of Complete Freund's Adjuvant (CFA)—induced inflammatory pain and employed network pharmacology to predict the potential therapeutic targets of ponicidin for pain treatment.

**Methods:**

Initially, we established a mouse model of inflammatory pain induced by Complete Freund's Adjuvant (CFA). Following the establishment of the model, the analgesic effects of ponicidin were assessed using behavioral tests, and further validation was conducted through hematoxylin and eosin (H&E) staining, enzyme-linked immunosorbent assay (ELISA), and immunofluorescence methods. Subsequently, we analyzed the potential analgesic targets of ponicidin using network pharmacology approaches and molecular docking.

**Results:**

In this study, we observed that ponicidin has a significant alleviating effect on CFA-induced inflammatory pain. Our results suggest that ponicidin may alleviate inflammatory pain by reducing inflammatory responses in the spinal cord and hind paw of CFA model mice. Furthermore, we found that ponicidin can mitigate the activation of macrophages in the subcutaneous tissue of the hind paw and microglia in the dorsal horn of the spinal cord. Network pharmacology analysis suggests that ponicidin may exert its analgesic effects through a multi-target, multi-pathway mechanism. Key transcription factors such as *nuclear factor NF-*κ*B p105 subunit* (*NFKB1*), *RELA, SP1, signal transducer and activator of transcription 3* (*STAT3*), and *repressor element 1 silencing transcription factor* (*REST*) may be involved in the underlying mechanisms of ponicidin's analgesic action. Through molecular docking and experimental validation, we have identified *toll-like receptor 4* (*TLR4*) and *hypoxia-inducible factor 1-alpha* (*HIF1A*) as key targets of ponicidin's analgesic effects.

**Conclusions:**

Ponicidin alleviates inflammatory pain by reducing inflammatory responses in the spinal cord and hind paw of the CFA model mice. *TLR4* and *HIF1A* may as key targets for the analgesic effects of ponicidin.

## Introduction

Pain is an inherently distressing sensory perception that frequently accompanies various pathological conditions, including tissue injury, infection, cancer, and inflammatory disorders. It is also a predominant symptom of numerous chronic diseases, such as diabetic neuropathy, rheumatoid arthritis, osteoarthritis, irritable bowel syndrome, and ulcerative colitis (1). The prevalence of chronic pain ranges from 11 to 40% (2), with the incidence of moderate to severe disabling pain estimated at 10.4 to 14.3% (3). Chronic pain imposes a significant economic burden on society. It is reported that approximately one-third of Americans suffer from chronic pain, resulting in annual medical expenses and productivity losses of 560 billion to 635 billion (4). Consequently, the management of chronic pain has become a formidable challenge for humanity.

Pharmacological intervention represents the primary approach to pain management, with non-steroidal anti-inflammatory drugs (NSAIDs) and opioids being the predominant therapeutic agents. However, these medications are associated with uncertain efficacy and numerous side effects. Consequently, there is an urgent need to develop analgesic drugs with both precise therapeutic effects and minimal side effects.

Ponicidin is a diterpenoid compound extracted and purified from traditional medicinal herbs such as *Rabdosia rubescens* and *Isodon japonicas* (5, 6). It exhibits various biological effects, including immunomodulatory, anti-inflammatory, antiviral, and antitumor properties (5–8). Pain is closely associated with immune responses, with neuroimmune interactions occurring at the peripheral, spinal, and central levels of the pain pathway (9). Extensive evidence supports the role of neuroinflammation in promoting peripheral and central sensitization, leading to pain hypersensitivity (10–12).

However, whether ponicidin can alleviate inflammatory pain through its anti-inflammatory effects and potential target molecules remains to be further explored. Consequently, in this study, we analyzed the analgesic effects of ponicidin in a Complete Freund's Adjuvant (CFA)-induced inflammatory pain mouse model. Additionally, we employed network pharmacology to predict the potential targets of ponicidin for pain treatment, which were subsequently confirmed through bioinformatics analysis. These findings hold significant implications for future investigations into the clinical potential of ponicidin.

## Materials and methods

### Animals and procedures

Male C57/BL6 mice (6–8 weeks old, weighing 18–20g) were purchased from the Animal Center of Southern Medical University (Guangzhou, China). The mice were housed in a Specific Pathogen-Free (SPF) facility with *ad libitum* access to food and water. They were randomly assigned to three groups (5 mice in each group): the sham surgery group, the CFA group, and the CFA + ponicidin group. An inflammatory pain model was established in mice by subcutaneous injection of CFA (20 μL, Sigma, St. Louis, MO), while the sham surgery group received an equivalent volume of saline. The CFA + ponicidin group received intraperitoneal injections of ponicidin (10 mg/kg, MedChemExpress, USA) at 8, 24, and 48 h post-CFA injection. On the third day following CFA injection, mice spinal cords or hind paw subcutaneous tissues were collected for experimentation. All animal studies were approved by the Experimental Animal Ethics Committee of the Experimental Animal Center at Southern Medical University (IACUC-LAC-20240618-008).

### Behavioral testing

Behavioral tests in mice were conducted 1 day before modeling and on days 1, 3, 5, and 7 post-modeling. Mice were acclimated to the testing environment for at least 2 days prior to baseline testing. The mechanic threshold of mice was assessed using the Von Frey monofilament test. Mice were placed in a box with an elevated metal mesh floor and allowed to acclimate for 30 min before testing. The Von Frey filament was applied perpendicularly to the plantar surface of the mouse's paw. Each mouse was tested three times, and the average threshold was taken, with a minimum 10-min rest period between each test. Thermal sensitivity was evaluated using the Hargreaves apparatus (Ugo Basile), which applies infrared heat to the plantar surface of the hind paw, measuring the paw withdrawal latency. A cutoff time of 20 s was set. The thermal test was repeated three times at 20-min intervals, and the average value was taken. Investigators were blinded to the behavioral group assignments.

### ELISA for cytokine level

The left hind paw subcutaneous tissues from mice of different treatment groups were lysed and cytokine levels were determined. The concentrations of *IL-1*β (Proteintech, KE10003), *TNF-*α (Proteintech, KE10002), and *IL-6* (Proteintech, KE10007) levels were measured by ELISA according to the manufacturer's instructions. Optical density (OD) measurements were taken at 450 nm using a microplate reader (Thermo Scientific).

### Hematoxylin and eosin staining

On the third day following CFA injection, the left hind paw subcutaneous tissues of mice were harvested for experimentation. Mice were anesthetized with isoflurane (2%) and sequentially perfused with 37°C saline and a 4% paraformaldehyde solution (pH 7.4; 4°C). The tissue was then immediately excised and fixed in 10% buffered formalin for 48 h to prepare for routine paraffin histological examination. Paraffin-embedded sections of 5 μm thickness from different groups were stained with H&E.

### Immunofluorescence staining

Samples of hind paw subcutaneous tissue and corresponding L4-5 spinal cord were sectioned into 20-μm-thick cryosections using a cryostat microtome. The tissue sections were fixed in 4% paraformaldehyde (Solarbio, China) at room temperature for 10 min, then permeabilized and blocked with 0.5% Triton X-100 (Sigma-Aldrich) and 3% bovine serum albumin (BSA, Solarbio, China) at room temperature for 1 h. Subsequently, the sections were incubated with diluted primary antibodies against *CD206* (1:500 dilution, Proteintech 18,704-1-AP), Iba1 (1:1000 dilution, Wako 559-24761), calcitonin gene-related peptide (*CGRP*) (1:100 dilution, Santa Cruz SC57053), *GFAP* (1:100 dilution, Proteintech 16,825-1-AP), and inducible nitric oxide synthase (*iNOS*; 1:200 dilution, Proteintech 18,985-1-AP). The sections were then incubated with appropriate secondary antibodies (1:500 dilution, Alexa Fluor 488-labeled goat anti-rabbit, mouse IgG, Jackson Immuno Research, West Grove, PA) at room temperature for 1 h. Finally, the slides were mounted with DAPI (4′,6-diamidino-2-phenylindole) containing anti-fade fluorescence mounting medium. Images were acquired using an upright manual fluorescence microscope (Zeiss, Imager D2, Germany) and a confocal laser scanning microscope system (Zeiss, LSM 980, Germany), and then processed using Adobe Photoshop 8.0 software (Adobe Systems, Mountain View, CA). Fluorescence images of *iNOS, CD206, CGRP*, and *IBA1* were semi-quantitatively analyzed using ImageJ software version 1.8.0 (National Institutes of Health, Bethesda, MA, USA).

#### Screening of potential drug and disease related targets

Pain-related targets were collated from four disease-associated databases: the Human Phenotype Ontology [HPO, http://www.human-phenotype-ontology.org; (13)], DisGeNET [https://www.disgenet.org; (14)], the National Center for Biotechnology Information (NCBI) Gene database, and the Pharmacogenomics Knowledge Base [PharmGKB; (15)]. Utilizing the keyword “pain,” we queried these databases for known pain-related targets, extracted the relevant data, and eliminated duplicate genes to compile a list of pain-associated targets.

Ponicidin-related targets were identified from two databases: the Chemistry and Biology (ChEMBL) database and the Target-Prediction protein-protein interaction (PPI) database. Utilizing the keyword “ponicidin,” we searched for associated targets, extracted the relevant data, and merged it to compile a list of ponicidin-related targets. Subsequently, in the UniProt (16) database, we specified “Homo sapiens” as the species of interest and standardized the retrieved target names to their corresponding gene nomenclature. We then employed Venny 2.1.0 (http://bioinfogp.cnb.csic.es/tools/Venny/index.html) to create a Venn diagram depicting the potential targets of ponicidin and pain. Subsequently, we utilized the network analysis and visualization platform Cytoscape 3.9.1 (https://cytoscape.org; Cytoscape: A Software Environment for Integrated Models of Biomolecular Interaction Networks—PubMed, n.d.) to visualize the relevant targets, thereby generating a graphical representation of the analgesic effects of ponicidin. Construction of PPI network and network topology analysis.

Utilizing the STRING database version 11.5 [https://cn.string-db.org; (17)], we searched for protein-protein interactions among the target genes, specifying Homo sapiens as the species and setting the “highest confidence” threshold to 0.7 to obtain the PPI data, excluding targets with a degree of zero. We then imported the PPI data into Cytoscape 3.9.1 software to construct the PPI network and performed topological analysis using the Network Analyzer (18).

#### GO enrichment analysis and KEGG pathway analysis

In this study, we conducted a Gene Ontology (GO) functional analysis of the common targets of ponicidin and pain using the DAVID database. Additionally, we employed an online bioinformatics tool (http://www.bioinformatics.com.cn) to perform KEGG [https://www.kegg.jp; (19)] pathway enrichment analysis on these target genes. Subsequently, we utilized Cytoscape 3.9.1 to analyze the top 10 nodes by degree in the PPI network and the top 20 KEGG pathways, thereby generating a network analysis map that identifies key pathways and targets.

#### Identification of the functional clusters of common targets of ponicidin and pain

In complex biological information networks, certain genes or proteins are closely related and share similar functions, allowing them to be considered as clusters that play a significant role in coordinating biological processes. The information associated with each node in the network can facilitate cluster analysis and the construction of functional modules (20). Metascape (http://metascape.org/) is a platform that employs the Molecular Complex Detection (MCODE) algorithm (20) to aggregate similar proteins and construct functional modules. By importing 84 targets into Metascape and utilizing the MCODE analysis, we identified functional modules within the PPI network. Furthermore, we predicted the transcription factors that regulate the common targets of ponicidin and pain. Additionally, we forecasted the diseases associated with these common targets.

### Molecular docking verification

Based on the findings, we selected the highest degree node in the PPI network as the receptor for docking with the ligand ponicidin. The structure of the target protein was retrieved from the Protein Data Bank (PDB; http://www.rcsb.org/) and subsequently processed and docked using the AutoDockVina software. The level of binding free energy was used as the evaluation standard of the binding degree of compounds. As a result, a binding energy ≤ −7.0 kJ/mol was used as the screening criterion. The results were then imported into the PyMOL software for visualization analysis.

### Cell culture and treatment

The BV2 cell line was sourced from Servicebio Biological Technology Co. Ltd (Wuhan, China). The immortalized mouse BV2 microglial cell line was cultured in Dulbecco's Modified Eagle's Medium (DMEM, Gibco, USA) supplemented with 10% fetal bovine serum (FBS, Gibco, USA) at 37°C in a humidified atmosphere containing 5% carbon dioxide. Upon reaching 50% confluence, the cells were treated with phosphate-buffered saline (PBS, Gibco, USA) or ponicidin and incubated for 24 h. Subsequently, the BV2 cells were rinsed with PBS and treated with lipopolysaccharide (LPS, Sigma, USA) at a concentration of 1 μg/mL for an additional 24 h.

### Cell viability assay

Initially, cytotoxicity assays were conducted to ascertain the non-toxic concentrations of the extract. A total of 1.0 × 10^5^ cells per well were seeded onto a 96-well plate and incubated at 37°C for 12 h. Subsequently, the cells were exposed to various concentrations of ponicidin (5, 10, 25, and 50 μmol/L) and incubated at 37°C for durations of 12, 24, and 48 h, respectively. The cell viability was assessed using the Cell Counting Kit-8 (Beyotime, China).

### RNA extraction and quantitative real-time polymerase chain reaction (qRT-PCR)

Total RNA was extracted from spinal cord tissue using Trizol reagent (Thermo Fisher Scientific, USA). The total RNA was reverse-transcribed into cDNA using a Vazyme reverse transcription kit (Vazyme, China). Expression of mRNA was assessed using the Hieff^®^ qPCR SYBR Green Master Mix (YEASEN, Shanghai, China) for real-time quantitative PCR (qRT-PCR). The qRT-PCR was performed on an ABI QuantStudio 6 Flex system (Applied Biosystems, USA). Primers for mouse genes were synthesized by RiboBio (Guangzhou, China), and their sequences are provided in Table 1.

**Table 1:** Sequence of primers.

**Primer sequences (5** ^**′**^ **-3** ^**′**^ **)**		
**Gene**	**Forward**	**Reverse**
*18S*	AGTCCCTGCCCTTTGTACACA	CGATCCGAGGGCCTCACTA
*TLR4*	ATGGCATGGCTTACACCACC	GAGGCCAATTTTGTCTCCACA
*HIF1A*	TCTCGGCGAAGCAAAGAGTC	AGCCATCTAGGGCTTTCAGATAA

### Validation of molecular docking results by qRT-PCR

Microglia in the spinal cord played a crucial role in pain modulation (21). We established a neuroinflammatory model by stimulating BV-2 microglia with LPS and employed qRT-PCR to verify whether Toll-like Receptor 4 (*TLR4*) and Hypoxia-Inducible Factor 1-alpha (*HIF-1A*), identified from the molecular docking results, are targets of ponicidin within this model.

### Statistical analysis

Statistical analyses were conducted using Prism software (GraphPad, San Diego, CA). Data are presented as mean ± standard error of the mean (SEM). For data obtained from behavioral tests, a two-way repeated measures analysis of variance (ANOVA) was employed, followed by Tukey's *post-hoc* test to analyze differences among groups. Data acquired from ELISA and immunofluorescence staining were analyzed using one-way ANOVA and Tukey's *post-hoc* test for multiple comparisons. The Shapiro-Wilk normality test confirmed a normal distribution of the data; hence, parametric tests were utilized for comparisons. A *p*-value of < 0.05 was considered to indicate statistically significant differences.

## Results

### Ponicidin alleviates complete Freund's adjuvant-induced inflammatory pain and suppresses inflammation

**Figure 1:**
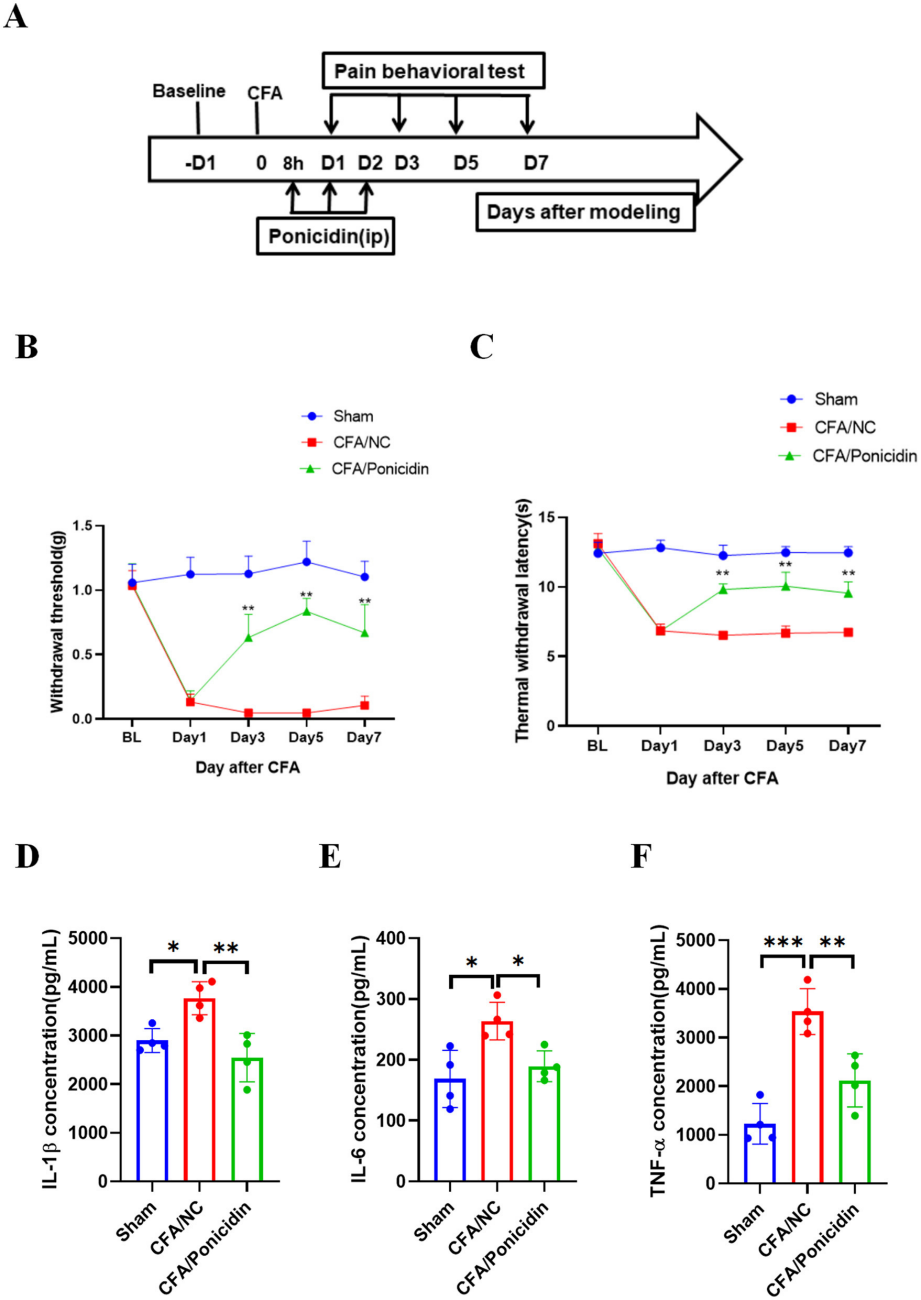
Ponicidin alleviates complete Freund's adjuvant-induced inflammatory pain and suppresses inflammation. **(A)** We established an experimental protocol for inducing inflammatory pain using CFA. **(B)** The 50% paw withdrawal threshold (PWT) was measured for mice in different treatment groups. Compared to the CFA group, the CFA + Ponicidin group exhibited a significantly increased mechanical threshold on days 3, 5, and 7 post-modeling (**p < 0.01, *n* = 5). **(C)** Thermal latency was assessed for the left hind paws of mice in different treatment groups. Compared to the CFA group, the CFA + Ponicidin group showed a significantly increased thermal latency on days 3, 5, and 7 post-modeling (**p < 0.01, *n* = 5). **(D–F)** ELISA was used to detect the protein levels of *IL-1*β **(D)**, *IL-6* **(E)**, and *TNF-*α **(F)** in the subcutaneous tissue of the mouse hind paws. Each group consisted of 4 mice. Data are presented as mean ± SEM. *p < 0.05, **p < 0.01, ***p < 0.001.

First, we established a mouse model of inflammatory pain by administering a subcutaneous injection of 20 μL of CFA into the hind paw of mice. Ponicidin (10 mg/kg) was administered intraperitoneally at 8, 24, and 48 h post-CFA injection (Figure 1A). The results demonstrated that the CFA + Ponicidin group exhibited significant anti-nociceptive effects compared to the CFA group on days 3, 5, and 7 post-modeling (Figures 1B, C). Compared to the CFA group, the CFA + Ponicidin group maintained a significant increase in mechanical threshold and thermal latency until day 7, although the analgesic effect began to decline from day 5 (Figures 1B, C). The results of the rotarod experiments indicate no significant differences in the mice's motor function (Supplementary Figure S1). These findings suggest that ponicidin can produce a potent analgesic effect in CFA mice in the short term.

On day 3 post-CFA injection, we collected hind paw tissue from mice to assess changes in inflammation-related cytokines. We found that, compared to the CFA group, the CFA + Ponicidin group showed a significant downregulation of IL-1β, IL-6, and TNF-α in the hind paw tissue (Figures 1D–F). Collectively, these results indicate that ponicidin can alleviate CFA-induced inflammatory pain and suppress inflammation in the paw tissue of mice.

### Ponicidin can significantly alleviate complete Freund's adjuvant-induced peripheral inflammation

**Figure 2:**
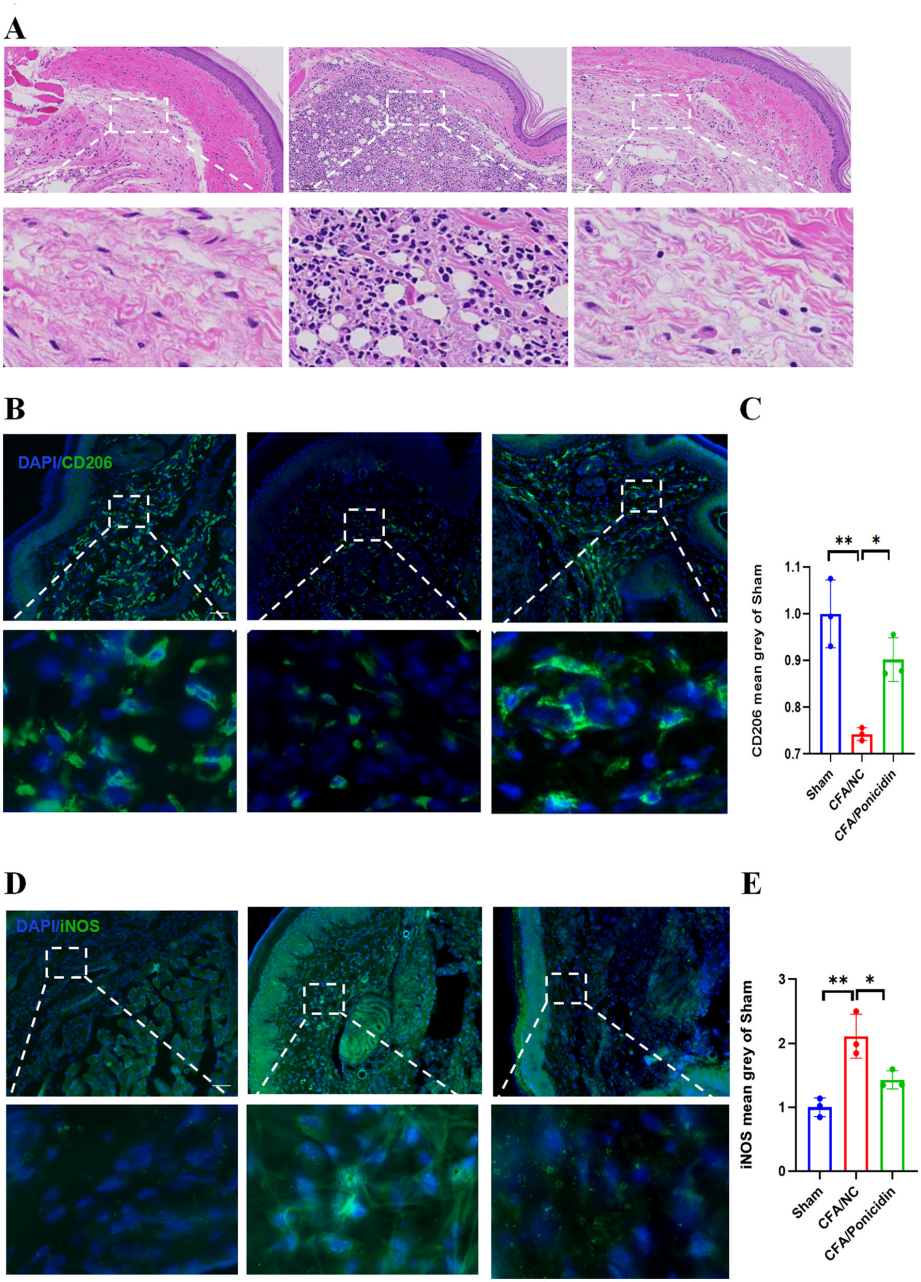
Ponicidin significantly alleviates complete Freund's adjuvant-induced peripheral inflammation. **(A)** H&E staining was conducted to assess tissue morphology. **(B)** Immunofluorescence analysis revealed the presence of *CD206*-positive cells (green) in the subcutaneous tissue of the hind paw from mice with CFA-induced inflammatory pain. DAPI nuclear staining is shown in blue (scale bar: 100 μm). **(C)** Quantitative analysis indicated a significant decrease in the mean grayscale value of *CD206* in the CFA/NC group compared to the sham and CFA/Ponicidin groups. Data are presented as mean ± SEM. *p < 0.05, **p < 0.01. **(D)** Immunofluorescence analysis showed the presence of *iNOS*-positive cells (green) in the subcutaneous tissue of the hind paw from mice with CFA-induced inflammatory pain. DAPI nuclear staining is shown in blue (scale bar: 100 μm). **(E)** Quantitative analysis demonstrated a significant increase in the mean grayscale value of *iNOS* in the CFA/NC group compared to the sham and CFA/Ponicidin groups. Data are presented as mean ± SEM. *p < 0.05, **p < 0.01.

H&E staining of mouse hind paw tissues revealed significant inflammatory infiltration and tissue structural damage in the subcutaneous tissues of the CFA group (Figure 2A). Compared to the CFA group, the CFA + Ponicidin group exhibited a marked reduction in inflammatory infiltration of the subcutaneous tissues. Subsequently, we analyzed the expression of *iNOS* and *CD206* in the mouse hind paw tissues. The expression of *iNOS* was significantly increased (Figures 2B, C), while the expression of *CD206* was significantly decreased in the hind paw tissues of the CFA group (Figures 2D, E). In contrast, the CFA + Ponicidin group showed a significant decrease in *iNOS* expression and a significant increase in *CD206* expression in the hind paw tissues (Figures 2B–E). These results indicate that ponicidin can significantly alleviate local subcutaneous inflammation in mice following CFA modeling.

### Ponicidin can significantly alleviate complete Freund's adjuvant-induced neuroinflammation at the spinal cord level

**Figure 3:**
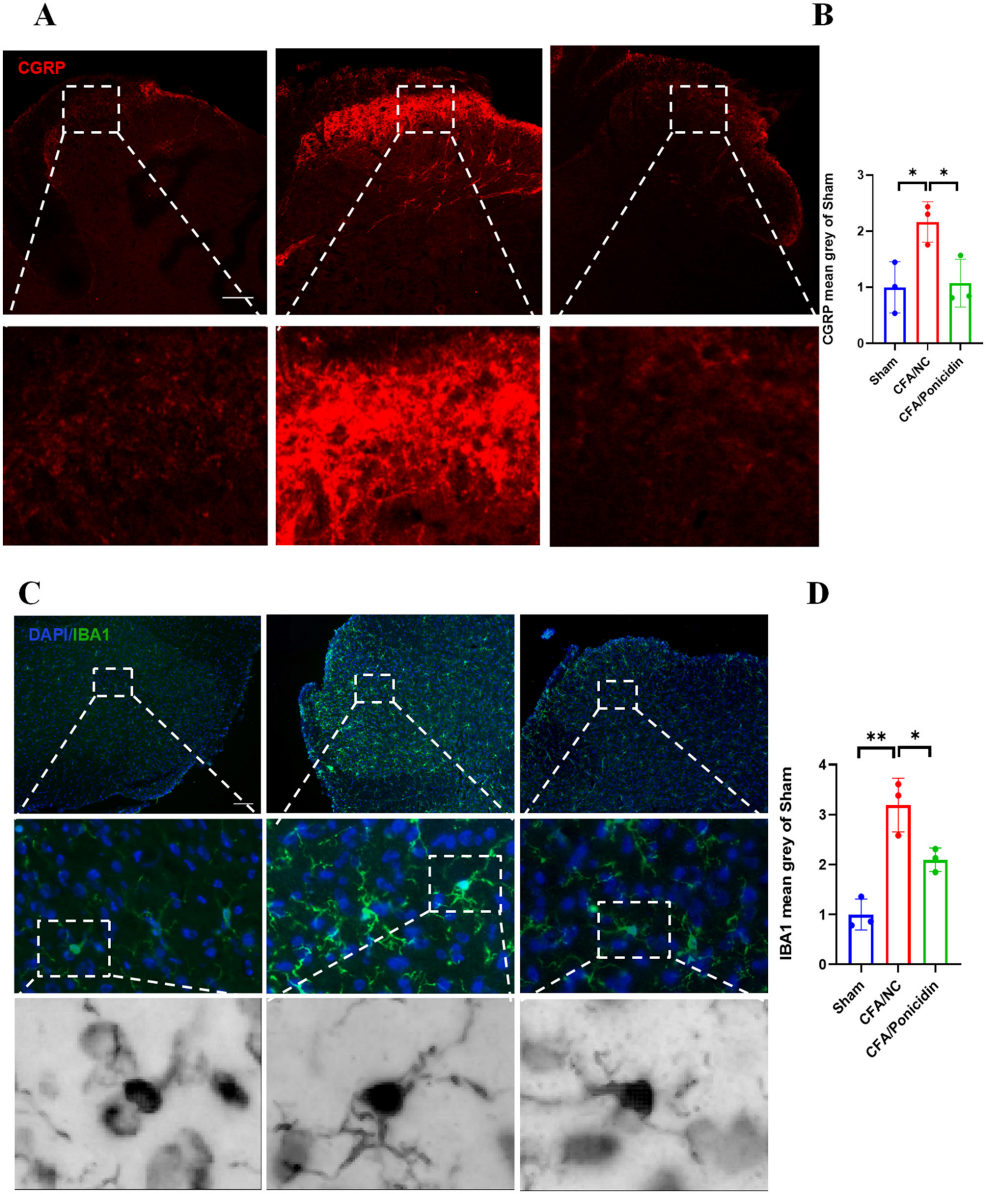
Ponicidin significantly alleviates complete Freund's adjuvant-induced neuroinflammation. **(A)** Immunofluorescence analysis revealed the expression of *CGRP* in the spinal cord dorsal horn of mice with CFA-induced inflammatory pain (stained red) (scale bar: 200 μm). **(C)** Immunofluorescence analysis demonstrated the presence of Iba1-positive microglia (stained green) in the spinal cord dorsal horn of mice with CFA-induced inflammatory pain (scale bar: 200 μm). **(B, D)** Quantitative results showed a significant increase in the mean grayscale values of *CGRP* and *Iba1* in the CFA/NC group compared to the sham and CFA/Ponicidin groups. Data are presented as mean ± SEM. *p < 0.05, **p < 0.01.

We investigated the impact of ponicidin on the production of *CGRP* in the spinal cord. Our findings revealed that compared to the CFA group, the CFA + Ponicidin group exhibited a significant decrease in *CGRP* levels in the dorsal horn of the spinal cord, suggesting that the antinociceptive mechanism of ponicidin may involve the inhibition of spinal *CGRP* production (Figures 3A, B). We also examined the impact of ponicidin on the activation of spinal neurons. Our findings revealed that compared to the CFA group, the CFA + Ponicidin group exhibited a significant reduction in spinal *cFos* levels (Supplementary Figure S2), suggesting that ponicidin may exert analgesic effects by suppressing the activation of spinal neurons. Subsequently, we analyzed the changes in *Iba1*-positive microglia in the L4-5 dorsal horn of the spinal cord. We observed that microglia in the L4-5 dorsal horn were significantly activated following the induction of inflammatory pain by CFA (Figures 3C, D). Compared to the CFA group, the number of activated microglia was markedly reduced in the CFA + Ponicidin group (Figures 3C, D). These results indicate that ponicidin may suppress spinal neuroinflammation by inhibiting the production of *CGRP* and the activation of microglia in the spinal cord of CFA mice.

### Common targets of ponicidin and pain

**Figure 4:**
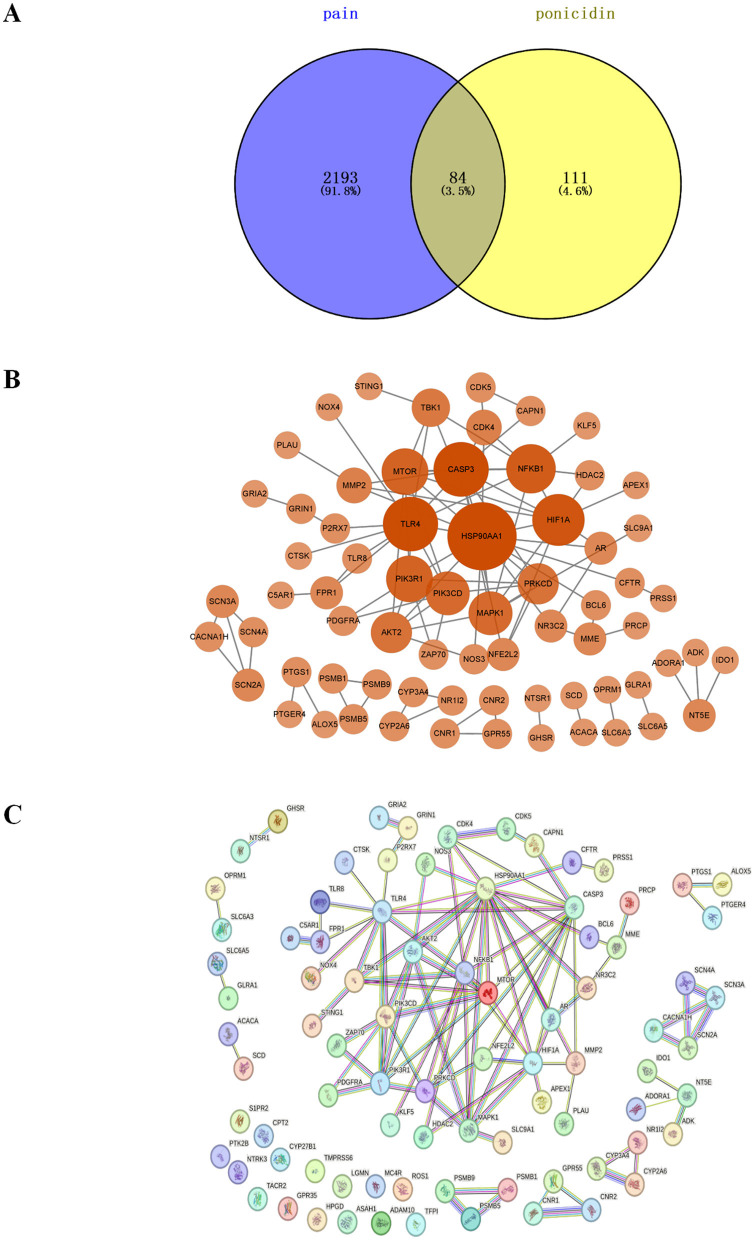
The plot of intersection and interaction between drug and disease targets and PPI network. **(A)** Purple represents targets of pain, yellow represents targets of ponicidin, and yellow-green represents targets of ponicidin comorbid pain. **(B)** After removing out the targets with a node degree of 0 and performing a topological analysis of the PPI network, the weight of the targets is represented by the size and color depth of the nodes. **(C)** PPI network analysis was performed on overlapping targets.

We identified 2,277 pain-related targets from four disease databases. From two traditional Chinese medicine databases, we obtained 195 drug targets associated with ponicidin. By intersecting these datasets using a Venn diagram, 84 common targets between ponicidin and pain were discovered (Figure 4A). These targets are considered candidate targets for the analgesic effects of ponicidin.

#### PPI network and topological analysis of common targets of ponicidin and pain

We constructed a PPI network and imported the PPI network of 69 core genes with a node degree greater than zero into Cytoscape for topological analysis (Figures 4B, C). The top 10 targets, listed in order of node degree, are Heat Shock Protein 90-alpha (*HSP90AA1*, node degree = 16), *Caspase 3* (*CASP3*, node degree = 11), *TLR4* (node degree = 11), *HIF1A* (node degree = 10), *Nuclear Factor NF-kappa-B p105* subunit (*NFKB1*, node degree = 9), *Serine/Threonine Protein Kinase mTOR* (*mTOR*, node degree = 8), *Phosphatidylinositol 3-kinase regulatory subunit alpha* (*PIK3R1*, node degree = 8) *Mitogen-Activated Protein Kinase 1* (*MAPK1*, node degree = 7), *Phosphoinositol-4,5-Bisphosphate 3-Kinase Catalytic Delta Isoform* (*PIK3CD*, node degree = 7), *AKT Serine/Threonine Kinase 2* (*AKT2*, node degree = 6), and *Protein Kinase C Delta* (*PRKCD*, node degree = 6). We hypothesize that these proteins are the core targets through which ponicidin exerts its significant therapeutic effects in pain management.

### GO and KEGG pathway enrichment analysis of common targets of ponicidin and pain

**Figure 5:**
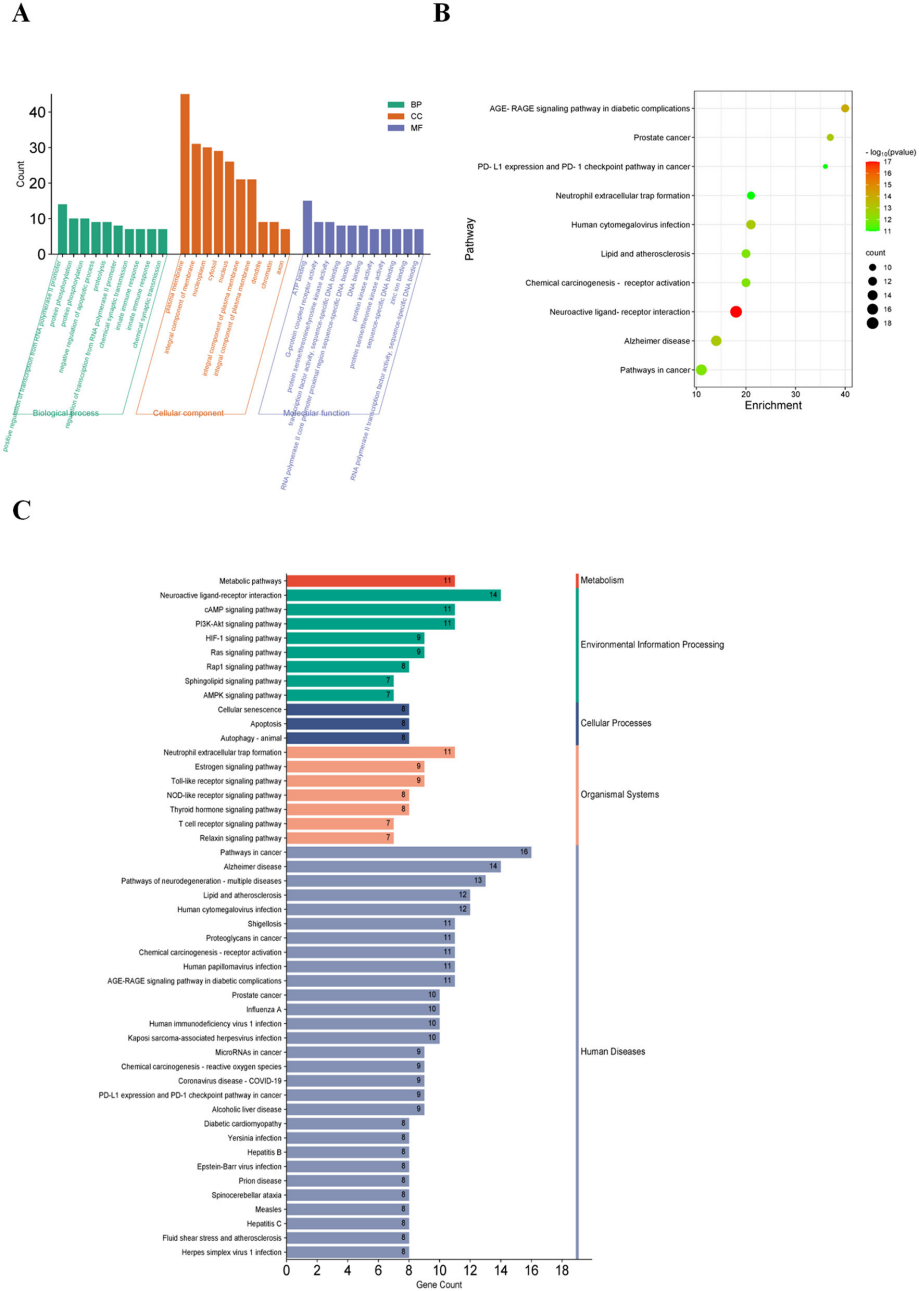
GO and KEGG analysis. **(A)** After GO and Metascape analysis of 84 key targets, the top 10 results of the two rankings were taken as the same. **(B)** KEGG pathways enriched in the Metascape platform (the size of the nodes indicates the number of enriched genes, and the color depth indicates the size of *p*-value). **(C)** Results of KEGG enrichment analysis (different colors indicate the different systems of action, and the pathways in each system are arranged in descending order by the number of enriched genes).

We performed functional annotation clustering on the common targets of ponicidin and pain using the DAVID database. Ponicidin was mainly enriched in response to positive regulation of transcription from RNA polymerase II promoter and protein phosphorylation in the biological process (BP) category. For cellular component (CC), ponicidin was mainly enriched in the plasma membrane, an integral component of the membrane, and nucleoplasm. For molecular function (MF), it was mainly enriched in ATP binding, G-protein coupled receptor activity, and protein serine/threonine/tyrosine kinase activity (Figure 5A).

**Figure 6:**
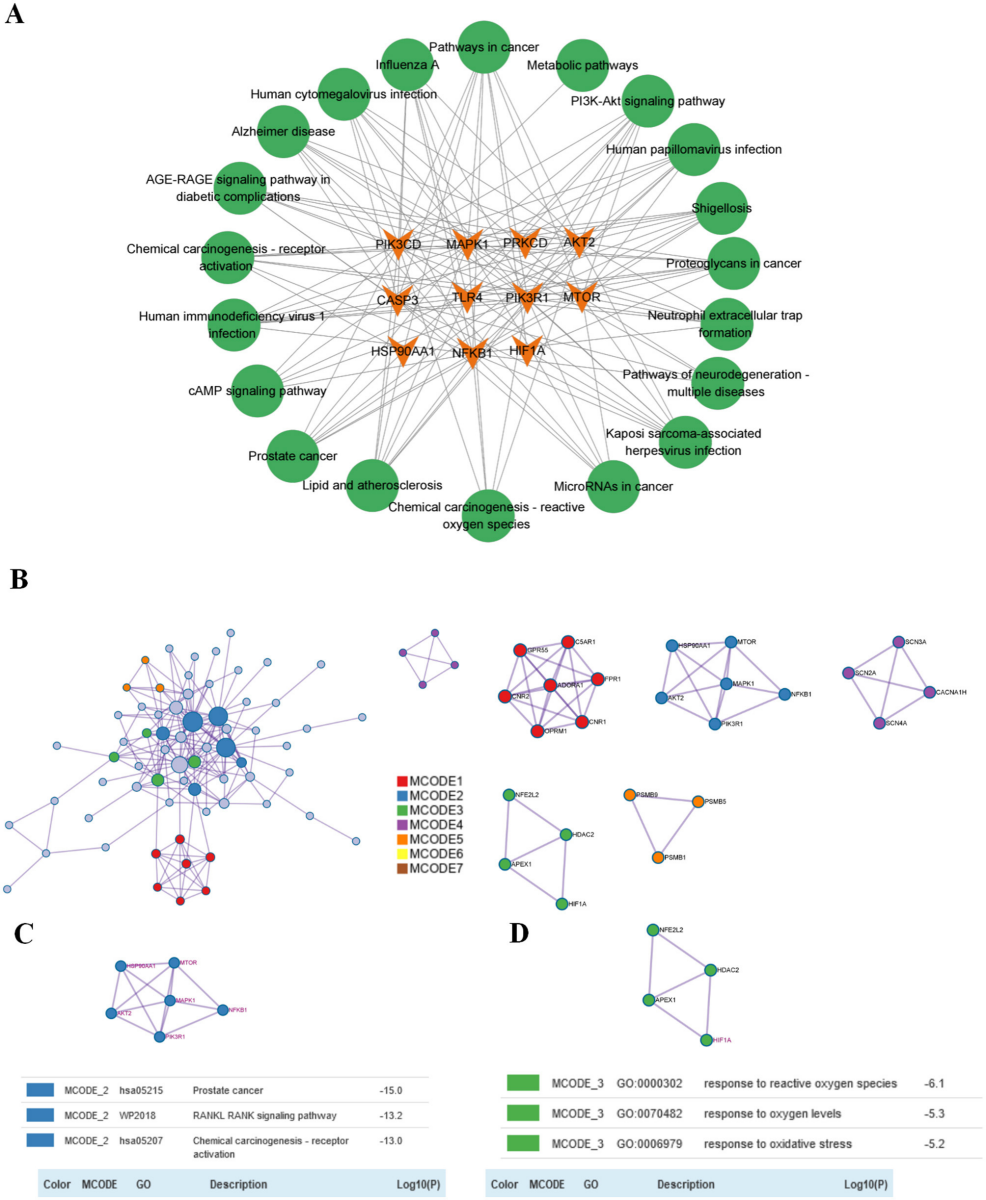
Enrichment network diagram of the top 10 (11 in total) core targets in the PPI network and KEGG pathways and MCODE cluster analysis and protein function module. **(A)** Intersection of the top 10 in KEGG enrichment analysis and the top 10 in PPI analysis. **(B)** MCODE cluster analysis was performed on 84 targets. **(C)** Pathway enrichment results for MCODE2. Gene names in purple indicate genes belonging to the top 10 node degrees (11 in total) in the PPI network. **(D)** Pathway enrichment results for MCODE3.

Additionally, 10 KEGG-enriched pathways were obtained after the intersection with Metascape (Figure 5B, pathways arranged by *p*-value) and KEGG database. In addition, KEGG enrichment pathways were obtained in the KEGG database (Figure 5C, taxonomic arrangement of pathways and number of enriched genes). Enriched pathways included Pathways in cancer, Alzheimer's disease, Neuroactive ligand-receptor interaction, and Pathways of neurodegeneration- multiple diseases. Subsequently, we employed Cytoscape 3.9.1 to visualize the relationships between the top 10 targets by node degree and the top 20 pathways (Figure 6A). Our analysis revealed that these genes are predominantly involved in pathways associated with inflammation and oxidative stress.

### MCODE analysis of common targets of ponicidin and pain

**Figure 7:**
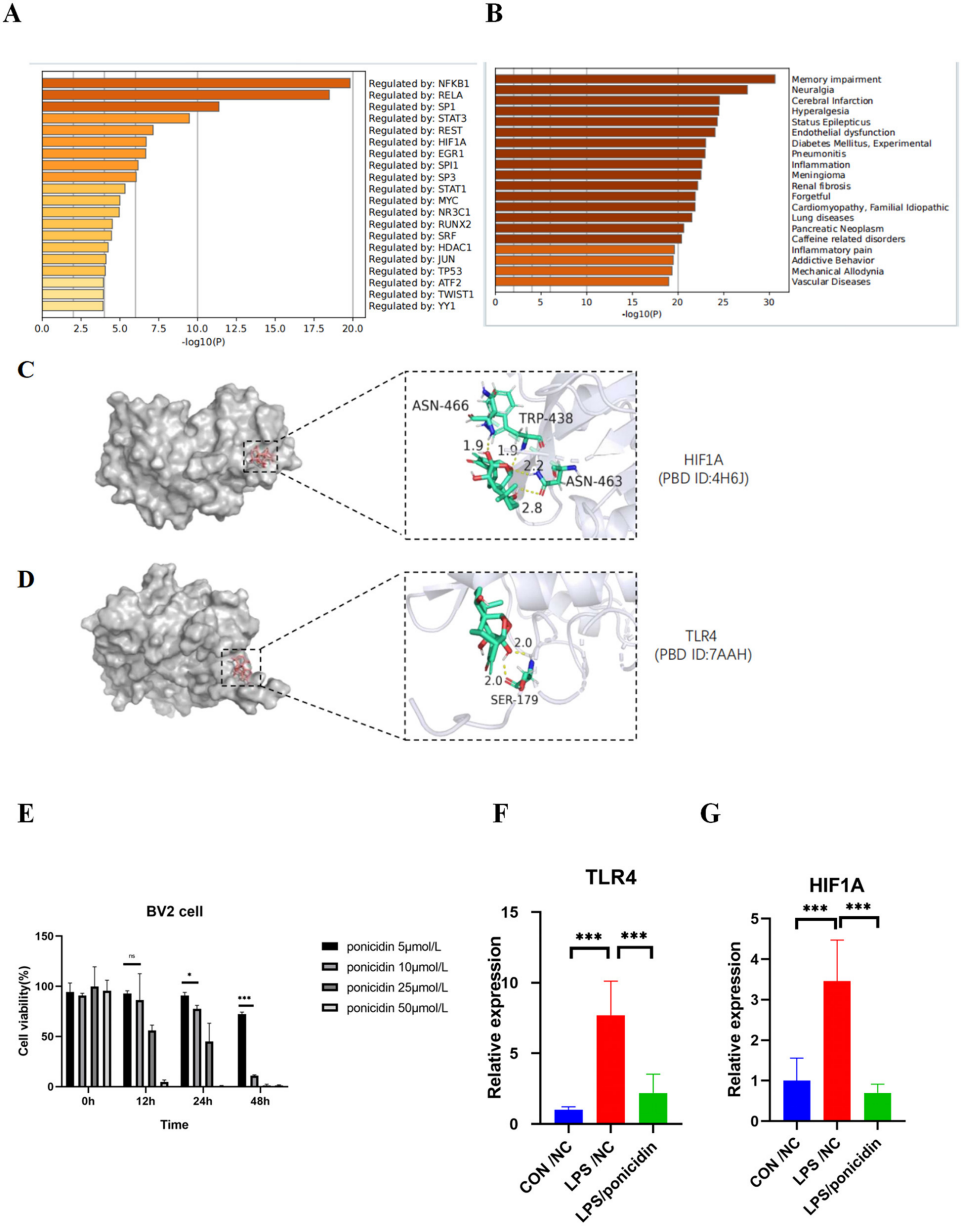
Protein function module and analysis of target-drug docking simulation and validation by qPCR. **(A)** Bar graph of transcription factors enrichment analysis (colored by *p*-values). **(B)** Summary of enrichment analysis in DisGeNET (colored by *p*-values). **(C, D)** Analysis of target-drug docking simulation. **(E)** Cytotoxicity profile of Ponicidin on BV-2 microglia. The data are presented as mean ± SD from three independent experiments. *p < 0.01 and ***p < 0.001. Two-way ANOVA followed by Tukey test was employed to determine the statistical differences between groups. **(F, G)** Effects of Ponicidin on the production of TLR4 and HIF1A in LPS-induced BV-2 microglia. The data are presented as mean ± SD from three independent experiments. ***p < 0.001. One-way ANOVA followed by Tukey test was employed to determine the statistical differences between groups.

We imported the 84 targets into Metascape for MCODE clustering analysis, yielding five functional modules (Figure 6B). Notably, the MCODE2 module (score = 2.0, depicted in purple in Figure 6C) encompasses six of the top 10 nodes by degree in the PPI network, while the MCODE3 module (score = 1.25, also depicted in purple in Figure 6D) contains one such node. This suggests a close association between these two functional modules and the analgesic effects of ponicidin. The pathways enriched in MCODE2 include prostate cancer, *RANKL-RANK* signaling pathway, and chemical carcinogenesis—receptor activation. The key genes involved are *HSP90AA1* (degree = 16), *NFKB1* (degree = 9), *mTOR* (degree = 8), *PIK3R1* (degree = 8), *MAPK1* (degree = 7), and *AKT2* (degree = 6). MCODE3 is primarily enriched in pathways related to the response to reactive oxygen species, oxygen levels, and oxidative stress, with *HIF1A* (degree = 10) identified as a key gene. Furthermore, we identified the top 10 transcription factors associated with ponicidin's analgesic effects, complete with *P*-values (Figure 7A). Utilizing Metascape, we also obtained ponicidin-associated disease phenotypes with *P*-values (Figure 7B). The top 10 enriched phenotypes related to ponicidin-induced diseases are predominantly linked to analgesics, with the target diseases ranked as follows: second for neuralgia, fourth for hyperalgesia, and ninth for inflammation (Figure 7B).

### Molecular docking verification

We imported the 3D structures of the top 10 targets from the PPI network into Autodock for docking with ponicidin. The binding energies of each target from the docking results are presented in Table 2. Ponicidin exhibited favorable docking outcomes with the corresponding proteins, with key amino acids primarily interacting through hydrogen bonds. The relatively low docking energy values suggest that the compound can stably bind to the receptor proteins and exert its effects. We selected TLR4 and HIF1A, which had the lowest binding energies, for visualization (Figures 7C, D) and subsequent experimental validation.

**Table 2:** Binding afnity of target-ponicidin in the treatment of pain via molecular docking.

**Target name**	**PDB ID**	**Binding energy (kcal/mol)**
TLR4	7AAH	−8.08
HIF1A	4H6J	−7.01
MAPK1	8AOJ	−6.94
HSP90AA1	5J80	−6.62
PRKCD	1YRK	−6.15
PIK3R1	5GJI	−6.11
NFKB1	7LFC	−5.85
MTOR	7SOQ	−5.74
AKT2	1O6L	−5.69
CASP	2DKO	−5.68

### Validation of molecular docking results by qPCR

To evaluate the outcomes of the systematic network pharmacology analysis, we employed qPCR to examine the mRNA expression levels of TLR4 and HIF1A, which were identified in the molecular docking results, within an acute inflammatory model induced by LPS stimulation in BV2 cells. The mRNA expression levels of TLR4 and HIF1A were significantly reduced in the ponicidin group compared to the LPS group (Figures 7E–G). The validation results were consistent with the predicted outcomes.

## Discussion

Ponicidin, a diterpenoid compound isolated from traditional Chinese herbs such as Rabdosia rubescens and Isodon japonicus, has been identified as a potential analgesic agent. However, its efficacy in treating pain patients remains to be elucidated. In this study, we observed that ponicidin has a significant alleviating effect on CFA-induced inflammatory pain. Our results suggest that ponicidin may alleviate inflammatory pain by reducing inflammatory responses in the spinal cord and hind paw of CFA model mice. Furthermore, we found that ponicidin can mitigate the activation of macrophages in the subcutaneous tissue of the hind paw and microglia in the dorsal horn of the spinal cord.

Previous literature has reported that the anti-inflammatory effects of ponicidin are associated with the inhibition of macrophages and the production of pro-inflammatory cytokines (22). Our findings indicate that ponicidin can effectively suppress the infiltration of inflammatory cells and the release of pro-inflammatory cytokines in the hind paw. Local inflammation is characterized by an increase in pro-inflammatory M1 macrophages and a decrease in anti-inflammatory M2 macrophages, leading to the secretion of a large number of inflammatory cytokines (23, 24). Our immunofluorescence results from the mouse hind paw show that ponicidin can effectively inhibit the expression of *iNOS* and increase the expression of *CD206* in the tissue. Our study suggests that ponicidin may suppress local inflammatory responses by modulating the ratio of M1/M2 macrophages in the hind paw tissue. *CGRP* is a member of the calcitonin peptide family and is predominantly distributed within the nervous system, particularly in nociceptive or primary afferent neurons (25). Extant literature documents the regulatory role of *CGRP* in various chronic pain conditions, with inhibition of *CGRP* alleviating pain (26, 27). *CGRP* is also known to upregulate the expression of pro-inflammatory factors such as *TNF-*α and *iNOS* (28). Our study indicates that following CFA-induced inflammatory pain, there is a significant increase in *CGRP* levels in the spinal cord dorsal horn, which can be markedly reduced by ponicidin. These findings suggest that ponicidin exerts its analgesic effect by inhibiting the release of spinal *CGRP*, and this action may be associated with the suppression of pro-inflammatory cytokine release.

Microglia in the spinal cord are crucial for the initiation and maintenance of inflammatory pain (29). Activation of spinal microglia triggers the release of pro-inflammatory factors such as *IL-1*β, *IL-6*, and *TNF-*α, which in turn further induce microglial activation (30). Furthermore, the activation of microglia can amplify the effects of pro-inflammatory factors and sustain central sensitization (31). Our research demonstrates that following CFA-induced inflammatory pain, microglia in the spinal cord dorsal horn are significantly activated. Notably, ponicidin can significantly suppress the activation of microglia in the spinal cord dorsal horn of mice with inflammatory pain. Therefore, ponicidin may alleviate inflammatory pain by reducing neuroinflammation through the inhibition of spinal microglial activation.

Our study suggests that ponicidin may inhibit inflammatory pain by suppressing the activation of macrophages in the hind paw tissue and microglia in the spinal cord. However, the mechanisms underlying the analgesic effects of ponicidin remain unclear. Thus, by employing network pharmacology methods, we identified and analyzed 84 potential targets of ponicidin for the treatment of pain. Among these, the top 11 targets were associated with various signaling pathways involved in inflammatory responses. These pathways encompass innate immunity, the *AGE-RAGE* signaling pathway, neutrophil extracellular trap formation, and the *PI3K-AKT* pathway. Previous research has demonstrated that the *RAGE* signaling pathway can induce neuroinflammation in neuropathic pain (32, 33). The involvement of the neutrophil extracellular trap formation signaling pathway in chronic pain has also been reported (34). These findings suggest that inflammation is a significant common phenotype in both pain and ponicidin. Metascape integrates biological information from multiple databases (35), and we utilized it to investigate the interactions and potential functions among common targets. The top 11 common targets, identified by node degree, were predominantly enriched in MCODE2 and MCODE3, which are associated with functions and pathways related to oxidative stress and cancer. The top 10 diseases enriched by the 84 targets related to ponicidin include Neuralgia, Hyperalgesia, and Inflammation. The top five enriched transcription factors among the 84 common targets are *NF-*κ*B1, RELA, SP1, STAT3*, and *REST*.

*RELA* (*NF-*κ*B p65*) and *NF-*κ*B1* (*NF-*κ*B p50*) are principal components of the *NF-*κ*B* family, forming the *p65/p50* heterodimer, which modulates inflammatory responses within the nervous system (36–38). Activation of *NF-*κ*B* induces the expression of *NLRP3* and pro-inflammatory mediators such as *IL-6, COX2*, and *TNF-*α, thereby promoting neuroinflammation and pain development (39). The *mitogen-activated protein kinase* (*MAPK*) pathway can modulate the *NF-*κ*B* pathway and is involved in the regulation of pain states (40).

Previous research has indicated that the transcription factor *Sp1* is involved in the regulation of neuropathic pain (41–43). In the spinal dorsal horn neurons of mice with the spinal nerve ligation (SNL) model, *Sp1* is highly expressed. Silencing *Sp1* has been shown to alleviate pain symptoms by downregulating *HDAC1* and *SOX10* (44). Sp1 contributes to the exacerbation of neuropathic pain by recruiting *HDAC2*, which inhibits the expression of *PGC-1*α, leading to dysfunction in spinal dorsal horn microglia and neurons (45).

The *Signal Transducer and Activator of Transcription 3* (*STAT3*) pathway plays a pivotal role in mediating inflammatory responses, and the activation of *STAT3* is significantly associated with the development of chronic pain. The *IL-6*/*STAT3* pathway has been shown to facilitate the formation of neuropathic pain and comorbid depression in spinal nerve injury (SNI) rats (46). In the spinal cord injury (SCI) model, *IL-6*-induced activation of the *JAK2/STAT3* signaling pathway in spinal dorsal horn microglia and astrocytes contributes to the progression of pain (47). Additionally, *TNF-*α aids in the activation of *STAT3* and the enhancement of neuronal excitability. *TNF-*α can directly or indirectly regulate gene expression through the *JAK/STAT3* signaling pathway, activating the *STAT3* pathway and inducing neuropathic pain (48–50).

The *neuronal restrictive silencer factor/repressor element-1 silencing transcription factor* (*NRSF/REST*) is a transcriptional repressor that plays a significant role in the chronic pain process. In a fibromyalgia model, *NRSF/REST* has been shown to reverse hyperalgesia or allodynia through epigenetic modifications (51). Another study demonstrated that the specific deletion of *REST* in dorsal root ganglia (DRG) effectively prevented the development of hypersensitivity in three distinct chronic pain models (CFA, SNI, and PSNL) (52). In the SNL model, *REST* in DRG neurons not only facilitates the transition from acute to chronic pain following nerve injury but also contributes to the inhibition of *Chrm2* and the reduction of muscarinic analgesia (53).

*TLR4* plays a pivotal role in innate immune responses within both the peripheral and central nervous systems. In the central nervous system (CNS), *TLR4* is predominantly expressed in microglial cells, where it primarily modulates the production of pro-inflammatory cytokines (54). *TLR4* is also expressed on primary sensory neurons and neurons expressing *CGRP* and *transient receptor potential vanilloid 1* (*TRPV1*) (55). *Damaged sensory neurons may release extracellular matrix molecules and damage-associated molecular patterns (DAMPs)*, which *TLR4* detects, activating immune cells and thereby influencing pain perception (54, 56). In our molecular docking results, *TLR4* exhibited the highest binding affinity for ponicidin (-8.08 kcal/mol). Given that *TLR4* is mainly distributed in microglial cells in the CNS, our findings indicate that BV2 cells show increased *TLR4* mRNA expression following LPS stimulation, and ponicidin is capable of reducing this upregulation, aligning with previous research findings.

In this study, we employed network pharmacology and molecular docking methods to predict the potential analgesic targets of ponicidin. Further *in vivo* and *in vitro* experiments are warranted to validate the analgesic targets and downstream pathways of ponicidin.

## Conclusions

In summary, ponicidin alleviates inflammatory pain by reducing inflammatory responses in the spinal cord and hind paw of the CFA model mice. The results from network pharmacology suggest that ponicidin may exert its analgesic effects through a multi-target, multi-pathway mechanism. Through molecular docking and experimental validation, we have identified *TLR4* and *HIF1A* as key targets of ponicidin's analgesic action.

## Data Availability

The original contributions presented in the study are publicly available. This data can be found here: https://www.jianguoyun.com/p/DW3kOtAQ15KbDRiqrfUFIAA.

## Supplementary material

Supplementary Figure S1 Ponicidin does not affect the motor function of mice with CFA-induced inflammatory pain. The results of the rotarod experiments indicate no significant differences in the mice's motor function.

Supplementary Figure S2 Ponicidin significantly alleviates complete Freund's adjuvant-induced *cFOS* level of spinal neurons. **(A)** Immunofluorescence analysis demonstrated the presence of *cFOS*-positive neurons (stained green) in the spinal cord dorsal horn of mice with CFA-induced inflammatory pain (scale bar: 100 μm). **(B)** Quantitative results showed a significant increase in the mean grayscale values of *cFOS* in the CFA/NC group compared to the sham and CFA/Ponicidin groups. Data are presented as mean ± SEM. ^*^*p* < 0.05, ^**^*p* < 0.01.
