# Supplementary material for: The effect of ponicidin on CFA-induced chronic inflammatory pain and its mechanism based on network pharmacology and molecular docking
Source: Front Med (Lausanne). 2025 Feb 19;12:1510271. doi: 10.3389/fmed.2025.1510271 (PMC11908685; doi:10.3389/fmed.2025.1510271)
Supplement: Supplementary Figure S1 — Ponicidin does not affect the motor function of mice with CFA-induced inflammatory pain. The results of the rotarod experiments indicate no significant differences in the mice's motor function. [file Data_Sheet_1.pdf]

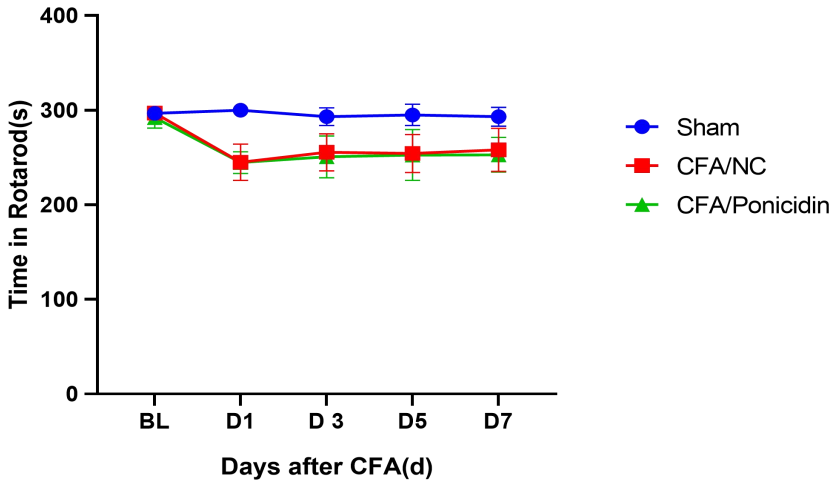

Fig S1 Ponicidin does not affect the motor function of mice with CFA-induced inflammatory pain. The results of the rotarod experiments indicate no significant differences in the mice's motor function.

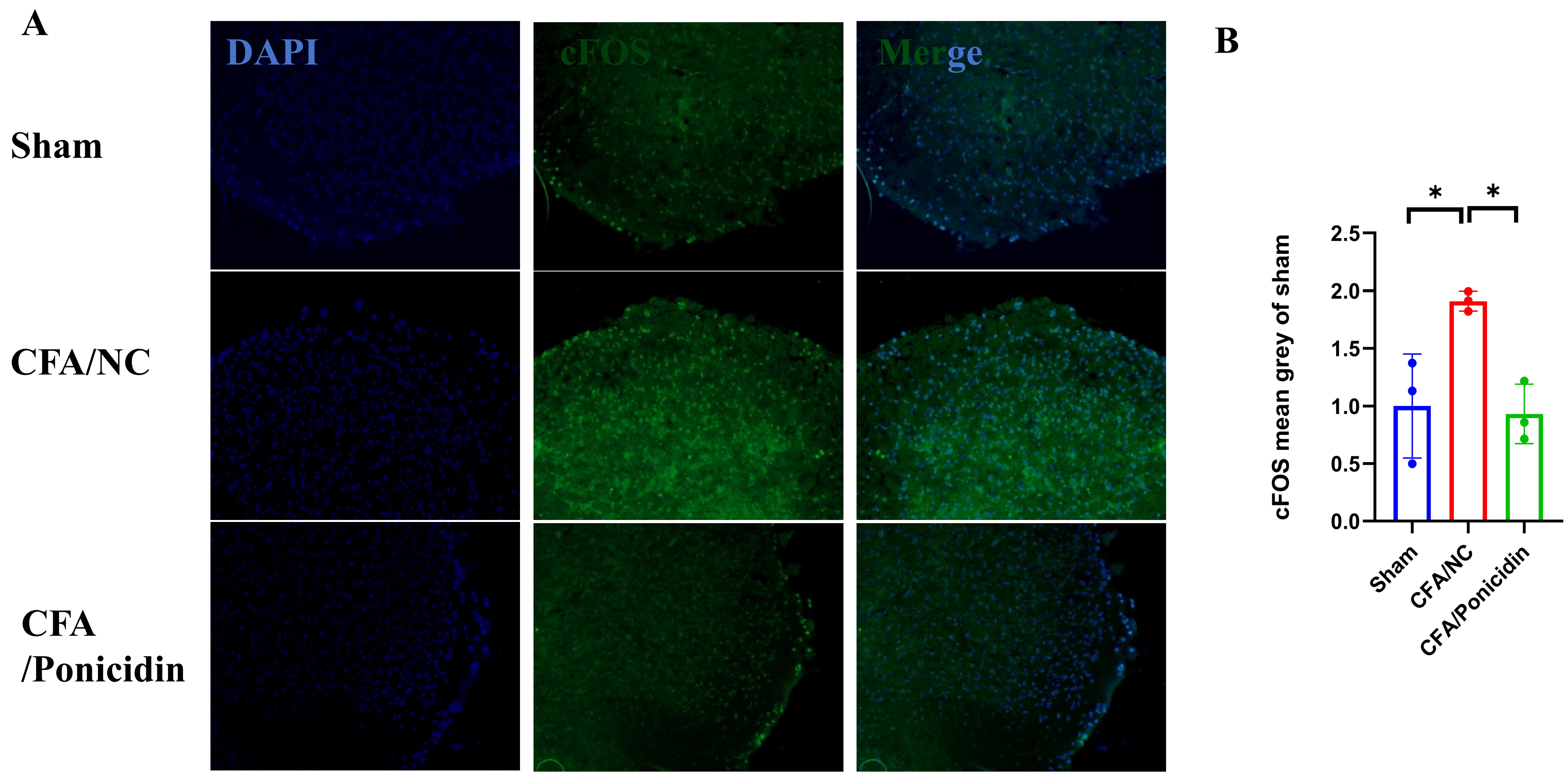

Fig S2. Ponicidin significantly alleviates complete Freund's adjuvant-induced cFOS level of spinal neurons.(A) Immunofluorescence analysis demonstrated the presence of cFOS-positive neurons (stained green) in the spinal cord dorsal horn of mice with CFA-induced inflammatory pain (scale bar: 100 μm).(B) Quantitative results showed a significant increase in the mean grayscale values of cFOS in the CFA/NC group compared to the sham and CFA/Ponicidin groups. Data are presented as mean ± SEM. *$p < 0.05$, **$p < 0.01$.
